# Predicting individual-level income from Facebook profiles

**DOI:** 10.1371/journal.pone.0214369

**Published:** 2019-03-28

**Authors:** Sandra C. Matz, Jochen I. Menges, David J. Stillwell, H. Andrew Schwartz

**Affiliations:** 1 Columbia Business School, Columbia University, New York, NY, United States; 2 Department of Business Administration, University of Zurich, Zurich, Switzerland; 3 Judge Business School, University of Cambridge, Cambridge, United Kingdom; 4 Department of Computer Science, Stony-Brook University, Stony Brook, NY, United States; Tilburg University, NETHERLANDS

## Abstract

Information about a person’s income can be useful in several business-related contexts, such as personalized advertising or salary negotiations. However, many people consider this information private and are reluctant to share it. In this paper, we show that income is predictable from the digital footprints people leave on Facebook. Applying an established machine learning method to an income-representative sample of 2,623 U.S. Americans, we found that (i) Facebook Likes and Status Updates alone predicted a person’s income with an accuracy of up to r = 0.43, and (ii) Facebook Likes and Status Updates added incremental predictive power above and beyond a range of socio-demographic variables (ΔR^2^ = 6–16%, with a correlation of up to r = 0.49). Our findings highlight both opportunities for businesses and legitimate privacy concerns that such prediction models pose to individuals and society when applied without individual consent.

## Introduction

Information about a person’s income is an asset in several business-relevant domains, such as marketing and recruiting. In the context of marketing, information about a person’s financial situation can be used to target consumers with the right products, e.g. through personalized advertising on platforms, such as Facebook or Google [1,2]. Which printer—ranging from $30 to $5,000—a company like Hewlett-Packard should advertise to a specific consumer depends partly on their financial means. While advertising a printer that is too expensive is likely to prevent consumers from buying it because they cannot afford it, advertising a product that is too cheap might prevent the consumer from buying it because they are accustomed to purchasing higher quality products. In the context of recruiting, information about a person’s current income can function as an anchor to help businesses negotiate (compare [3]). For example, knowing that a candidate’s current salary is in the ballpark of around $70k allows hiring managers to make a first offer that is ambitious yet plausible.

At the same time, people often do not wish to share information about how well off they are financially. Among users of online dating platforms, for example, less than 10% provide information about their income [4]. Similarly, discussing one’s salary with friends and co-workers is highly uncommon. Although the National Labor Relations Act of 1935 granted employees the right to disclose their salaries in the US, the cultural norm still considers it “inappropriate” ([5]; p. 261). In the words of a commentator in *The Atlantic*, “Asking a coworker about pay seems akin to asking about their sex life” [6]. This illustrates that people often consider information about their financial means private.

There are good reasons why people might be reluctant to disclose income to others. Consider the two business cases above. In the context of marketing, income targeting can not only be used to help people find the right products but also to exploit their vulnerabilities. For example, expensive pay-day loans could be targeted at individuals who have the biggest need for financial support, yet the least resources to afford an expensive loan [7]. Similarly, the knowledge of a candidate’s current salary might help the employer achieve a better negotiation deal, but it is also likely to perpetuate existing inequalities. For example, all else equal, a job candidate with a current salary of $80k is likely to be offered a higher salary for a new role than a job candidate with a current salary of $60k. Given that women–and many minority groups–have traditionally earned less for equal work [8], asking job candidates about their past salaries may reinforce the existing gender pay gap [9]. In late 2017, several major US cities responded to this problem by passing legislation that bars hiring managers from asking job candidates about their current or past salary [10].

However, barring potential employers from directly inquiring about a candidate’s financial situation in a job interview or hindering Facebook and Google from offering income-based targeting is unlikely to be enough to prevent businesses from obtaining this information. Income can be estimated indirectly from a job candidate’s or customer’s socio-demographic profile [11], their zip code [12], or their non-verbal cues in a job interview [13]. In addition, both employers and marketers today have access to a powerful and easy-to-access source of information: The Web and social media.

Whether it is their Facebook or Twitter accounts, their browsing logs, or the data captured by their smartphones, the digital profiles people create of themselves–both intentionally and unintentionally–are often extensive. On Facebook alone, there are more than 510,000 comments posted, 293,000 statuses updated, and 136,000 photos uploaded every minute [14]. Research shows that these digital footprints often reveal more about their owners than first meets the eye. Computers have accurately predicted people’s intimate socio-psychological characteristics, such as their personality, political ideology, or sexual orientation, from digital footprints including Facebook profiles [15–18], Twitter messages [19], personal websites [20], or pictures posted online [21,22].

A recent study suggests that the same idea holds true for the prediction of people’s financial situations: participants’ Twitter profiles accurately predicted their occupation-based income [23]. However, this study estimated income from participants’ self-reported job title, which considerably limits the conclusions that can be drawn from these results. For example, a person who reported to be an accountant was assigned the average income of an accountant as indicated by the Annual Survey of Hours and Earnings released by the Office for National Statistics of the UK. Consequently, this occupation-based income measure is a rather rough proxy of participants’ actual income that does not capture income variations within professions (e.g. an accountant working for a major strategy consultancy is likely to have a higher income than a self-employed accountant offering advice to small and medium-sized companies). What is missing is an analysis that goes beyond group-level income estimates and tests whether digital footprints can accurately predict a person’s income on the individual level. Can we tell how much a person earns based on the traces they leave on the Internet?

The current paper applies machine learning to test whether digital traces on Facebook can be used to predict a person’s self-reported income on the individual level. Although self-report measures are not without drawbacks (e.g. [24]), this much more nuanced and fine-grained assessment of income allows us to estimate the predictive power of digital footprints more accurately. We further test the generalizability of automatic income predictions by investigating new types of digital footprints: Facebook Likes and Status Updates. Although Facebook profiles merely constitute one of many digital traces of behavior (see discussion), they have been shown to provide highly intimate information about their owners [16]. In addition, they are particularly relevant to business contexts as they are a source that marketers and recruiters are known to consult in their decision-making process [25,26]. Considering the accuracy of the predictions reported in this paper and the cost required to create them (e.g. with regards to data science capabilities and data access), we return to the two use cases of marketing and recruiting in the discussion to estimate the likelihood that income predictions from digital footprints would be put into practice for each of them.

## Method

### Participants

**Fig 1:**
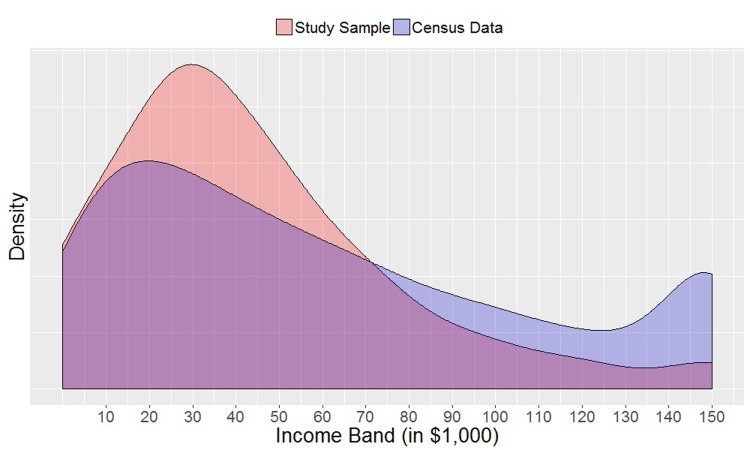
Density distributions of annual income. The distribution of the study sample is displayed in red, the distribution of the US Census data in blue.

We recruited a gender and income representative sample of US Facebook users through a paid Qualtrics panel in early 2015 (data was collected in line with Qualtrics’ and Facebook’s terms of service). A total number of 7,180 participants responded to the survey. We excluded all participants who (i) answered the attention check question incorrectly (N = 2,461), (ii) had used a Facebook ID that appeared in the dataset more than once (N = 516), or (iii) who had fewer than 10 Likes or 500 words in their Status Updates (N = 1,580). This left us with a total of 2,623 participants. Similar to the demographic distribution of Facebook users in the US [27], the average age of participants in our sample was 35.9 years (SD = 11.0), and 60% of participants were women, suggesting that our sample remained largely representative despite the relatively high attrition (see income distribution in Fig 1). All participants were fully informed of the data they would be sharing when consenting to participate. The study was approved by the University of Cambridge Department of Psychology Ethics Committee.

### Measures and procedure

Participants were asked to complete a 5-minute long questionnaire, which included questions about their zip code, age, gender, ethnicity, educational attainment, occupation industry, and personality as measured by the BFI-10, an established short measure of the Five Factor Model of personality [28]. In addition, they were asked about their annual income (“What was your total income in USD, before taxes and other deductions from January 1st 2014 to December 31st 2014? Please include money from jobs or self-employment, net income from business, farm or rent, pensions, dividends, welfare, social security payments, and any other money income that you received during that time”). Fig 1 displays the density distribution of participants’ self-reported income, which is compared to the density distribution of the US population in 2014 reported by the US Census Bureau. Due to the nature of our sample and the fact that we had to exclude a considerable number of participants (see section above), our income distribution deviates from the population distribution in that it is skewed towards low-income participants, even though it follows the same pattern.

At the beginning of the questionnaire participants were asked to provide their informed consent and to grant us access to their Facebook Likes (official Page Likes, i.e. excluding any other Likes such as friends’ photos or status updates) and Status Updates. On average, people included in our final dataset had 822 Likes (SD = 1,470) and 18,377 words in their Status Updates (SD = 30,132). Compared to previous publications using Facebook data (e.g. obtained from databases such as myPersonality.org which contains data from 2007 to 2012, [29]), the number of Likes and words in status updates is significantly higher. This is expected, however, as Likes and Status Updates accumulate over time and have seen an exponential growth in recent years. A person, who back in 2012 had 250 Likes, for example, is likely to have liked a substantial number of additional Pages over the following years, while deleting Likes remains rare.

### Prediction models

We developed three behavior-based prediction models based on: (i) Likes, (ii) language from Status Updates, and (iii) the combination of Likes and Status Updates. Following the procedure described by [15] for language-based assessment and an adapted version of [18] for Likes-based assessment, we built the prediction models in three steps.

In the first step, we turned Likes and Status Updates data into two large matrices—users by Likes and users by words—with users in rows and Likes/words in columns. For the user-Likes matrix, entries were set to 1 if the participant was associated with the Like and to 0 if the participant had no association with the Like. For the user-Status-Update matrix, we first extracted all words and phrases (up to 3 word sequences; e.g. “hit me up”) using a social media tokenizer [17], a computer program that automatically identifies typical words as well as emoticons, hashtags (e.g. “#election2016”), and slang (e.g. “g’nite”, “smh”–shaking my head). Entries were subsequently set to represent the relative frequency of word use, as well as binary 0/1 indicating whether the word was used at all. Function words, such as articles or pronouns, were retained intentionally as they have been found to significantly relate to individual differences. For example, “the” and other articles have been found to be used more frequently by those with higher intelligence or education [17,30] while self-references, such as “me,” have been found more frequent among individuals who are depressed or anxious [30,31].

In the second step, we applied singular value decomposition (SVD; [32])–a commonly used dimensionality reduction method–to reduce the two matrices to 300 dimensions for Likes and 700 dimensions for Status Updates (dimension based on past work; [15–16]). Because we have nearly 100,000 dimensions as initial input, SVD along with a Ridge penalty (discussed next), is used to avoid overfitting (e.g. [15]). To increase the robustness of our SVD solution, we developed the SVD matrices on a much larger sample of Facebook users that was available through the myPersonality dataset [29]. This step allowed us to deal with the sparsity of both Likes and Status Updates data in our sample (i.e. there were more than 855,000 unique Likes, and more than 32.8 million unique words and phrases, many of which were associated only with a handful of participants). In the third step, we ran ridge regression models (LASSO; [33]) to predict logged income from the two low dimensional matrices obtained in step two. Ridge regression is a widely-used machine learning algorithm that applies an L2 penalty to avoid overfitting when one has many independent variables.

To avoid overfitting in our model validation, we evaluated the accuracy of our model using separate training samples to fit our models, and hold-out samples to test their predictive power. Such cross-validation procedures including hold-out samples are the gold standard in predictive modeling. They address the problem that traditional in-sample methods (e.g. variance explained as expressed by R^2^) are highly biased when there is a large number of predictors. In fact, with tens of thousands of predictor variables, one would likely achieve near-perfect prediction accuracy in-sample, but this accuracy is unlikely to generalize to new samples [33].

Specifically, we used a standard 10-fold cross-validation procedure [34]. First, we randomly divided the dataset into ten equal-sized partitions (folds). In 10 iterations, we subsequently trained the LASSO model using data from nine of the folds (training set), and then tested for accuracy over the remaining hold-out fold (testing set). Once the out-of-sample predictions were made for all of the ten folds, we estimated the accuracy of our model by calculating Pearson’s Product-Moment correlation between the actual and predicted income values.

We further tested the predictive power of behavior-based predictions by establishing its incremental predictive validity above and beyond the following baseline socio-demographic and psychological comparisons: (i) demographics (gender, age, and ethnicity), (ii) education (8-point scale ranging from “Less than high school” to “Doctorate degree”), (iii) industry (24 industries adapted from the North American Industry Classification System), (iv) zip code income, (v) personality, and (vi) all variables from (i) to (v) combined. We ran significance tests to formally establish whether the added predictive power significantly improved the predictive accuracy of our model.

## Results

Table 1 displays the Pearson Product-Moment correlations for all the models we calculated. Likes alone predicted income with an accuracy of r = .27, and Status Updates predicted it with an accuracy of r = .41. Combining Status Updates and Likes further increased the predictive accuracy of all models to an accuracy of r *=* .43 for the model including both Likes and Status Updates. While far from being perfectly accurate, this correlation is considered a strong effect-size in the context of behavioral/psychological factors [35] and is higher than the accuracy of similar approaches used to predict personality factors [15].

**Table 1:** Pearson Product-Moment correlations between predicted and actual income values obtained from 10-fold cross-validated LASSO models. Column 1 displays model accuracies when using the psychological and socio-demographic controls only. Columns 2–4 display the accuracies of the model when adding Likes, Status Updates, and the combination of the two to the control models. Row 1 displays the accuracies for the models using Facebook data exclusively with no controls. All correlations are significant at p < 0.001.

	Controls	+ Likes	+ Status Updates	+ Likes & Status Updates
No controls	-	0.27	0.41	0.43
Personality	0.14	0.27	0.41	0.42
Demographics	0.16	0.32	0.43	0.43
Zip code Income	0.21	0.30	0.43	0.43
Industry	0.23	0.28	0.41	0.42
Education	0.30	0.34	0.44	0.44
All socio-demographics	0.42	0.42	0.48	0.48
All socio-dem. + personality	0.43	0.43	0.48	0.49

Both the language-based and Likes-based models provided incremental predictive accuracy over the socio-demographic and psychological baseline variables. Adding Facebook Likes and Status Updates to the socio-demographic controls increased the variance explained between 10% (when compared to education) and 16% (when compared to personality). Even when taking the most comprehensive baseline of all socio-demographic and personality variables–which together explained 18% of the variance–adding Facebook Likes and Status Updates increased variance explained by 6% to a total of 24% (r = 0.49). Notably, the incremental accuracy was mostly driven by Status Updates, with Facebook Likes adding none or little accuracy to the baseline models.

**Fig 2:**
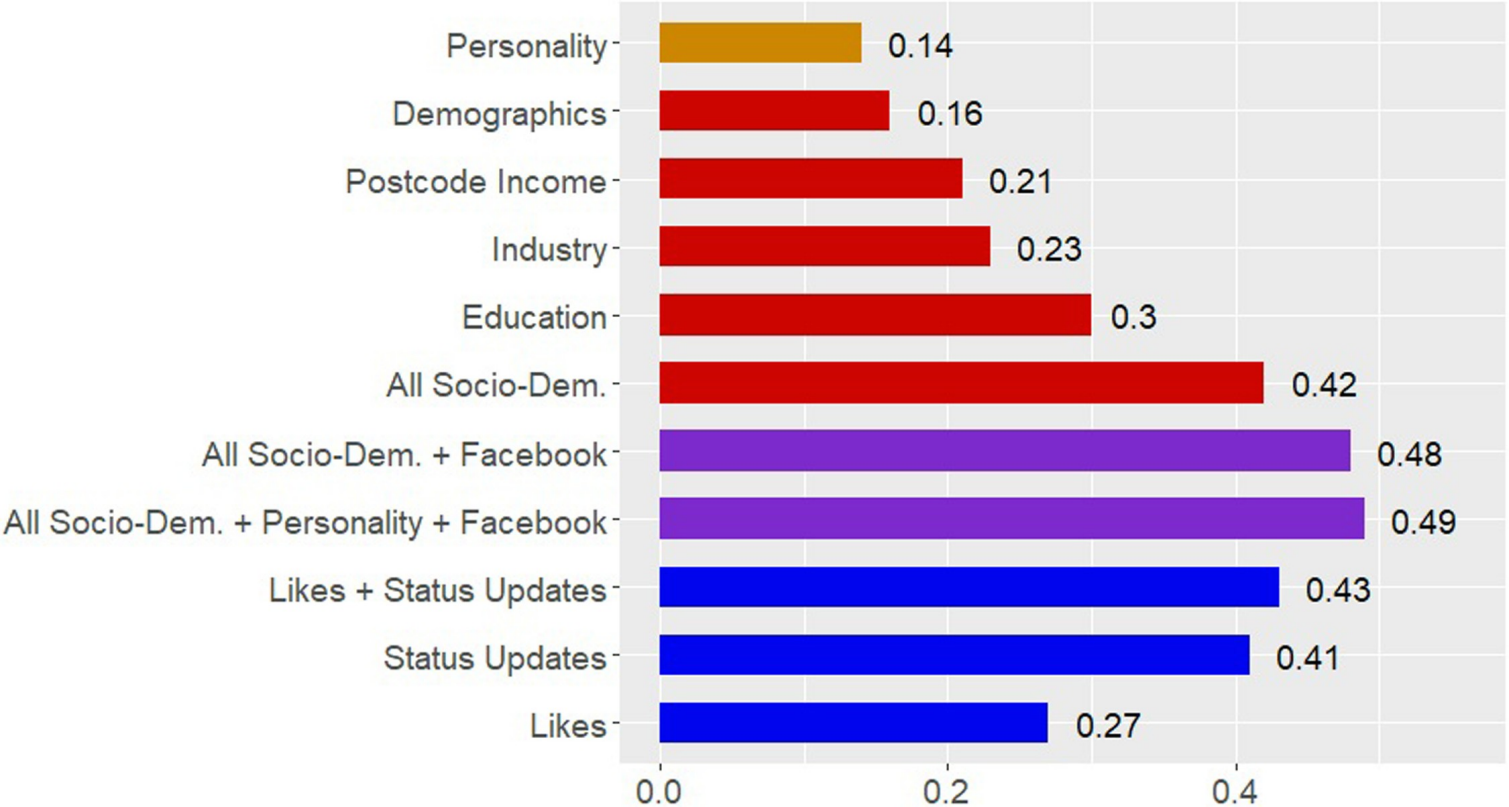
Pearson Product-Moment correlations between predicted and actual income values. Red bars indicate the predictive power of the socio-demographic variables used as baseline comparisons. The ‘Demographics” model includes age, gender, and ethnicity. Blue bars indicate the predictive accuracy of Facebook data, separated by Likes, Facebook Status updates, and a combination of the two. The purple bars display the results of the comprehensive models, which include both socio-demographic variables, personality and Facebook data.

Fig 2 displays Pearson Product-Moment correlations between the predicted and actual income values obtained for the Likes and Status Updates-based prediction models (blue bars). To provide a baseline comparison for evaluating the predictive power of our models, we also included the accuracies obtained from predictions made with psychological and socio-demographic baseline comparisons (and orange and red bars). Finally, we included the accuracy of the model combining all socio-demographic variables, psychological variables, and Facebook data (purple bars).

To develop a better understanding of the relationships between Facebook profiles and income, we looked at the individual words (or phrases) and Likes that were most strongly associated with low and high income. Table 2 shows the 10 Likes that displayed the highest positive correlations with income (right) and the Likes that displayed the highest negative correlations with income (left), when controlling for age and gender. Table 1 reveals that the Likes of high and low income individuals are markedly different: While the Likes associated with high income refer to concrete expensive travel and retail brands (e.g. “The Cosmopolitan of Las Vegas,” “Paula’s Choice,” or “Janie and Jack”), the Likes associated with low income refer to luxury but in a highly abstract and generalized way (e.g. “Amazing Things”) and often contain entire phrases (e.g. “Dentist Stop Talking to Me, I Cant Talk Your Hand is in My Mouth,” or “if i text a person in the same room as me, i stare at them 'til they get it”).

**Table 2:** Low and high income likes. Likes most strongly associated with high income (right) and low income (left), controlling for age and gender.

Low income	High income
❖ if i text a person in the same room as me, i stare at them 'til they get it	❖ The Smith Center
❖ We act like its a secret drug deal when someone is just giving us gum	❖ Sheets
❖ All Things Tumblr	❖ The Cosmopolitan of Las Vegas
❖ Funniest Pics	❖ Frankie J
❖ Amazing Things	❖ Beauty4Moms
❖ Eminem	❖ Janie and Jack
❖ Don't EVER break a pinky promise. That stuff is LEGIT.	❖ Paula’s Choice
❖ Bullet for my Valentine	❖ It Works Skinny Wrap Team
❖ Dentist Stop Talking to Me, I Cant Talk Your Hand is in My Mouth	❖ Pier 39
❖ Having a "sweatpants, hair tied, chillen with no makeup on" day	❖ X Out

**Fig 3:**
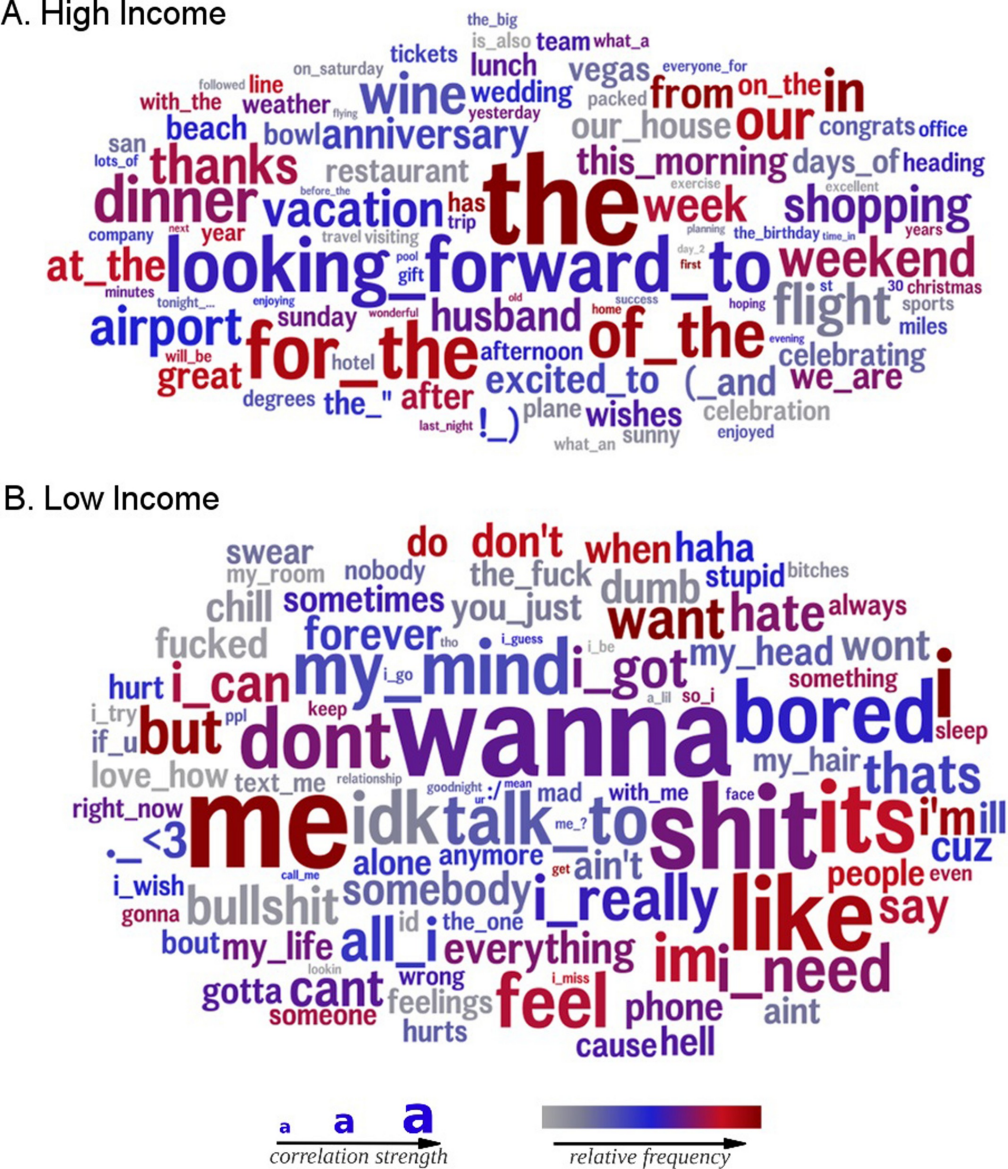
Low and high income word clouds. Words and phrases most positively correlated with income (top) and most negatively correlated with income (bottom), after controlling for age and gender.

Fig 3 displays two differential word clouds with the words most strongly associated with high income (top) and low income (bottom), controlling for age and gender. All words displayed are significantly related to income. Similar to the Likes, the differences in topics are remarkable and paint a clear and poignant picture. High income individuals talk about vacations (e.g. vacation, flight, beach, Vegas, airport) and pleasant activities that usually require spending a considerable amount of money (e.g. shopping, celebrating), express positive emotions (e.g. excited to, great), and use future-oriented words and phrases (e.g. looking forward to, afterwards). Low income individuals, on the other hand, are highly self-focused (e.g. I need, I can, I got, me), use colloquial language (e.g. idk, cuz), express negative feelings (e.g. hurt, hate, bored), and use more swear words and emoticons.

## Discussion

Our findings suggest that a person’s income can be predicted from their Facebook profile data. By obtaining access to individuals’ Facebook Likes and Status Updates (with their informed consent) along with a self-report of their person income, we were able to automatically predict personal income with relatively high accuracy (r = 0.43) based on the language and likes. The accuracy level was comparable to what we could achieve using a person’s comprehensive socio-demographic profile including age, gender, ethnicity, zip code level income, education, and industry (r = 0.42). Notably, integrating Facebook Likes and Status Updates with those socio-demographic as well as personality added incremental predictive accuracy to our baseline models (ΔR^2^ between 6% and 16%). As expected, the added value was strongest when considering relatively generic socio-demographic variables, such as age, gender, and ethnicity, but became marginal when considering all socio-demographic and psychological control variables at the same time.

Even though around 75% of the variance in income remained unexplained by our models, the prediction accuracy reported in this paper is high enough to be useful in contexts such as targeted marketing that do not require an extremely high degree of accuracy at the individual level. Yet, they do not seem to be accurate enough to establish a person’s income to the extent that it would be useful in the negotiation of starting salaries. Although we could distinguish between a person with a very low income and one with a very high income, our model is not sufficiently accurate to make fine-grained distinctions between a person making $70k or $75k a year. However, there is reason to believe that the accuracy of prediction models like ours could be further increased by using more robust and reliable income measures (e.g. actual instead of self-reported salary), as well as a combination of additional external (e.g. their LinkedIn, Facebook and Twitter profiles) and internal (e.g. their CV and application materials) data sources.

Importantly, the ability to build predictive models such as the one we introduced in this paper is not limited to a small number of researchers or companies that control huge datasets of personal information (e.g. Facebook or Google). Although it is not trivial to collect the necessary data and generate the predictive models that turn it into insights about a person’s income, our approach could be replicated by any company that receives access to both consumers’ digital footprints and their income information and that has reasonable data science capabilities. This barrier makes it unlikely for small to medium-sized businesses to integrate such predictions into their hiring decisions, as the costs do not outweigh the benefits. For example, employers could receive comparable insights into a candidate’s current income by simply asking them about their “expected salary”–an intervention that comes at no cost. For targeted marketing, however, there is no simple or obvious alternative, making it more likely that larger retail companies will engage in building similar technologies to improve their sales strategy In addition, there is a growing number of companies entering the market of predicting consumer insights from digital footprints (e.g. IBM Watson personality insights). The same way that fintech companies are already using people’s digital footprints to determine their credit scores [36], other companies might soon offer income predictions from a broad variety of digital sources as part of their portfolio, thereby making it easy for companies to benefit from such predictions without having to train and deploy their own machine learning models. As predictive technologies like the one described in this paper become more prevalent and easily accessible, it becomes paramount to carefully consider and publicly discuss their potential opportunities and challenges.

On the one hand, the ability to predict a person’s income unobtrusively from their digital footprint could offer opportunities that help both businesses and consumers if deployed in the right way. As we have outlined in the introduction, businesses might tailor their services and offers to the purchasing power of the individual consumer. Such personalization strategies directly benefit businesses by allowing them to target more accurately, and they could also benefit the consumer by suggesting products that fall into a realistic price (and quality) range, thus alleviating the risk of overspending. In addition, the information about a person’s income–or more broadly, their socio-economic status–could be used to increase the fairness of hiring decisions. As more and more companies transfer to an automatic assessment and screening of job candidates that is driven by computer-based algorithmic choices [37], it becomes paramount to ensure that these algorithms do not discriminate against specific subpopulations (e.g. women, minorities, low-income individuals, [38]). Because algorithms are trained on historical data, they are prone to formalizing and perpetuating existing biases [38,39]. For example, if women have been historically hired at a lower rate than men, then the algorithm would conclude that being “male” is a strong predictor of being “successful.” In order to actively overcome such biases, it is necessary to have the socio-demographic information about candidates that one aims to reduce biases against. That is, businesses cannot account for biases against traditionally underprivileged groups if they do not know whether a job candidate is a women or a man, or whether the candidate comes from a low or high socio-economic background. While managers can no longer legally–and should not ethically–ask their applicants about their previous income or socio-economic standing directly, estimating this information and using it in the context of auditing and de-biasing prediction models and decisions indirectly (without revealing it to the manager), could increase the fairness of hiring processes as long as the data processing was strictly controlled so that it does not influence the manager.

On the other hand, together with the larger body of literature on predicting highly intimate characteristics from digital footprints (e.g. [15,16]), our findings demonstrate the need for ethical guidelines for predictive technologies, as well as regulations on a policy level. Such guidelines and regulations are needed to protect people’s privacy and to ensure that these new technologies are used in the best interest of individuals and society at large. In most parts of the world–including the US–our approach to predicting a person’s income from his or her Facebook profile could be implemented without the knowledge of users. Although some of the more progressive data protection regulations, such as the European General Data Protection Regulation (GDPR), consider the implications of “profiling” and regulate its application (e.g. by requiring transparency and consent), they do not prohibit them entirely. There remains a substantial degree of freedom in how businesses implement predictive technologies like the one described in this paper; this makes an ongoing public and political discussion on the ethical challenges of those technologies paramount.

Taken together, the current research showed that Facebook Likes and Status updates not only predict self-reported income with the same degree of accuracy as standard socio-economic variables, but they also added incremental predictive power. The accuracy of these predictions is high enough for low stakes applications such as targeted marketing where outcomes are usually measured at the group level. However, but they are–in their current form–high enough for applications such as recruiting and salary negotiations, where outcomes are measured at the individual level and where there is a simple alternative to making a prediction. While providing opportunities for businesses to improve their services and aim for algorithmic fairness in their decisions, our findings also raise several critical questions regarding privacy and data protection. Progressive data protection regulations such as the GDPR alleviate those concerns to some extent, but they may not fully prevent the technology presented in this paper from being used in a way that is potentially harmful to people. For example, job candidates might feel the pressure to check a box saying they agree to their data being processed, even though they consider it invasive. It is therefore critical that businesses adhere to ethical principles and carefully consider both the potential costs and benefits before implementing income predictions from digital footprints. In addition, consumers need to be made aware of the possibilities that predictive technologies hold, and the fact that social media data (and other digital footprints) can reveal a lot more about them than they might think. Together with giving them the power to decide who uses their data, and for what purposes, this will allow them to decide for themselves whether they are willing to forsake their privacy in order to benefit from better services, or whether they consider the risks of potential abuse as being too high.
